# Serum Granulysin Levels in Vitiligo and Alopecia Areata: A Potential Biomarker for Disease Activity and Dermoscopic Evaluation

**DOI:** 10.3390/jcm14196894

**Published:** 2025-09-29

**Authors:** Hayam Ali AlRasheed, Amira Aboelmakarem Korkor, Yasmina Ahmed El Attar, Rowida Raafat Yousef, Mostafa M. Bahaa, Zainab Abdel Samad Ibrahim

**Affiliations:** 1 Department of Pharmacy Practice, College of Pharmacy, Princess Nourah bint Abdulrahman University, Riyadh 11564, Saudi Arabia; 2 Dermatology and Venereology Department, Faculty of Medicine, Tanta University, Tanta 31527, Egypt; amira.korkor@med.tanta.edu.eg (A.A.K.);; 3 Biochemistry Department, Faculty of Medicine, Tanta University, Tanta 31527, Egypt; 4 Pharmacy Practice Department, Faculty of Pharmacy, Horus University, New Damietta 34517, Egypt; 5 Pharmay Practice Department, Faculty of Pharmacy, Mansoura National University, Gamasa 7731168, Egypt

**Keywords:** granulysin level, vitiligo, alopecia areata, dermoscopy

## Abstract

**Background:** Vitiligo is a chronic, progressive skin disorder characterized by the development of sharply demarcated depigmented patches due to the loss of melanocytes. Alopecia areata (AA) is an autoimmune condition that presents with sudden, non-scarring hair loss affecting the scalp or other body areas. **Objective:** To evaluate serum granulysin (GNLY) levels in patients with vitiligo and AA to explore its potential role in the pathogenesis and activity of both diseases. **Methods:** A total of 80 participants were included: 65 patients and 15 healthy controls. Patients were divided into four groups: active vitiligo (n = 25), stable vitiligo (n = 25), active AA (n = 15), and a control group (n = 15). Serum GNLY levels were measured and analyzed in relation to clinical and dermoscopic features. **Results:** No significant correlation was found between GNLY levels and either age or Vitiligo Area Scoring Index in vitiligo patients. However, serum GNLY levels showed a significant association with the Vitiligo Disease Activity score. GNLY levels did not correlate with sex or the starburst pattern. In contrast, significant associations were found between elevated GNLY levels and dermoscopic signs of activity, including ill-defined lesion borders, satellite lesions, perifollicular pigmentation, and loss of pigment network. Both vitiligo and AA patients exhibited significantly higher GNLY levels than controls, with the highest concentrations observed in the active vitiligo group. **Conclusions:** The significant rise in serum GNLY levels in vitiligo and AA suggests a pathogenic role, with higher levels in active vitiligo indicating its potential utility as a biomarker for monitoring disease activity.

## 1. Introduction

Vitiligo is the most prevalent skin depigmentation disorder, marked by a gradual depletion of active melanocytes in the epidermis of affected areas. It commonly affects both the skin and hair, resulting in distinct, sharply defined white patches. Estimates of its global prevalence in the general population range from 0.06% to 2.28% [1]. The exact cause of vitiligo remains unclear, though it is widely recognized as a multifactorial condition influenced by a combination of genetic predisposition and environmental or non-genetic trigger [2]. Several mechanisms have been proposed to explain vitiligo pathogenesis, including autoimmune responses, insufficient melanocyte-stimulating factors, self-induced enzymatic damage, and aberrant neurogenic signals that may cause segmental melanocyte loss [3,4].

Alopecia areata (AA) is a persistent, immune-driven condition marked by sudden, non-scarring hair loss, which can vary from small, defined patches on the scalp to total hair loss on both the scalp and body [5]. It is the second most frequent cause of hair loss, after androgenetic alopecia [6]. Cytotoxic T lymphocytes (CTLs) are primarily responsible for the destruction of anagen hair follicles, which is a key factor in the development of AA [7]. The precise mechanisms by which inflammatory T cells trigger hair loss in alopecia areata remain unclear. In AA-affected hair follicles, elevated levels of cytotoxic T-cell mediators, including perforin, granzyme, Fas ligand, and granulysin (GNLY), are observed [8].

Although vitiligo and alopecia areata present with distinct clinical features, they share important immunopathogenic mechanisms [9]. Both are T cell–mediated autoimmune disorders in which cytotoxic CD8^+^ T lymphocytes and natural killer (NK) cells target pigment-bearing cells—melanocytes in vitiligo and melanocyte-containing hair follicle cells in AA [9]. These immune effector cells release cytolytic proteins, including perforin, granzymes, and GNLY, which mediate apoptosis and tissue destruction [10]. Moreover, both conditions exhibit overlapping cytokine profiles (e.g., IFN-γ, IL-15) and genetic susceptibility loci, supporting the concept of a shared autoimmune basis [11]. Considering these similarities, evaluating GNLY levels in both diseases within a single study may provide valuable insight into its role as a common biomarker of disease activity and pathogenesis.

Granulysin (GNLY) is a cytolytic protein stored in the granules of activated cytotoxic T lymphocytes and natural killer (NK) cells. Upon immune activation, GNLY is released into the extracellular space, where it induces apoptosis of target cells, including melanocytes in vitiligo and melanocyte-associated follicular cells in alopecia areata [12]. GNLY acts through pore formation and the initiation of mitochondrial apoptosis pathways, amplifying tissue injury and inflammation. Importantly, GNLY can be detected not only within lesional tissue but also in the circulation, reflecting systemic cytotoxic immune activity [13]. Serum levels therefore provide a clinically useful, minimally invasive biomarker that integrates localized and systemic immune responses. While skin biopsy can demonstrate local expression, serum sampling allows for serial monitoring, better patient compliance, and broader applicability in clinical practice [14].

Serum levels of GNLY are a reliable indicator of the activity of cell-mediated cytotoxic immune responses [15]. Increased expression and levels of GNLY in both tissue and serum have been previously observed in various diseases [16]. GNLY has been implicated in tissue destruction through mitochondrial damage and apoptosis. Elevated levels of GNLY have been observed in various autoimmune conditions [17], but its role in vitiligo and AA remains insufficiently explored. Considering the involvement of cytotoxic T lymphocytes in both diseases and the ability of GNLY to reflect cytotoxic immune activity, investigating serum GNLY levels may provide insight into its potential as a biomarker for disease pathogenesis and activity.

The primary endpoint of this study was to compare serum granulysin (GNLY) levels among patients with vitiligo, patients with alopecia areata, and healthy controls, in order to determine whether GNLY is elevated in these autoimmune pigmentary diseases. The key secondary endpoints were: (1) to assess the association between serum GNLY levels and disease activity (active vs. stable disease) within each patient group; (2) to evaluate correlations between serum GNLY and clinical severity indices and (3) to explore whether GNLY could serve as a shared biomarker across both diseases, potentially contributing to personalized patient monitoring.

## 2. Materials and Methods

This study involved 80 participants recruited from the Tanta University Hospital from January 2022 to August 2023. Institutional Review Board of Tanta University’s Faculty of Medicine amended the study protocol on 25 December 2021 and approved it after reviewing it for all ethical and academic issues (Approval Code: 35089/12/21). The study was conducted in compliance with the Helsinki Declaration and its revisions.

### 2.1. Study Design

Patients were divided into four groups: active vitiligo group (n = 25), stable vitiligo group (n = 25), control group (n = 15): healthy subjects (Matching the age and sex as vitiligo patients) and active AA group (n = 15): active alopecia areata was defined by the presence of progressive hair loss or new patches developing within three months [18].

### 2.2. Inclusion Criteria

Patients were eligible for enrollment if they were aged six years or older and had a clinical diagnosis of either vitiligo or AA established by an experienced dermatologist according to standard clinical criteria. For vitiligo, active vitiligo was defined as the occurrence of new depigmented patches or enlargement of pre-existing lesions within the preceding three months [19,20], whereas stable vitiligo was defined as the absence of new lesions or progression for at least six months [19]. For AA, active disease was defined as the appearance of new patches or progression of existing lesions during the last three months [18]. Healthy controls were age- and sex-matched individuals without any personal or family history of vitiligo, AA, or other autoimmune or chronic inflammatory skin diseases. All participants, or their legal guardians in the case of minors, were required to provide written informed consent and demonstrate willingness to comply with study procedures

### 2.3. Exclusion Criteria

Patients were excluded if they had any concomitant autoimmune, systemic inflammatory, or infectious diseases that could influence immune biomarkers. Individuals who had received systemic corticosteroids, immunosuppressive agents, or phototherapy within the three months preceding enrollment were not eligible, as these treatments might affect serum granulysin levels. Patients with a history of malignancy, metabolic disorders such as uncontrolled diabetes or thyroid disease, or significant cardiovascular, hepatic, or renal impairment were also excluded. Pregnant or lactating women were not included in the study. In addition, individuals who were unable or unwilling to provide informed consent, or who had incomplete clinical data, were excluded from participation. We excluded patients on recent systemic or topical immunomodulatory therapies, active infections, malignancy, or other systemic inflammatory/autoimmune diseases because these conditions and treatments can alter cytotoxic lymphocyte activity and circulating GNLY levels [21,22], potentially confounding the association between GNLY and disease activity in vitiligo and AA.

All patients were subjected to history taking, general and dermatological examinations (for evaluation of signs of activity (Ill-defined border, Satellite lesion, Starburst appearance [23,24]) and evaluation of vitiligo disease activity (VIDA) score and Vitiligo Area Scoring Index (VASI) score for all vitiligo patients.

### 2.4. Study Outcomes

#### 2.4.1. Primary Outcomes

The primary endpoint was the difference in serum GNLY levels between patient groups (vitiligo and AA) and healthy controls.

#### 2.4.2. Secondary Outcomes

The secondary endpoints of the study included the evaluation of the association between GNLY and disease activity by comparing levels in patients with active versus stable disease. In addition, the correlation between serum GNLY levels and validated severity indices such as VASI, and VIDA score for vitiligo, was assessed to examine the relationship with disease severity. Finally, the study explored whether GNLY elevation represented a shared biomarker across both vitiligo and AA, thereby reflecting potential common autoimmune and immunopathogenic mechanisms underlying the two conditions. Dermoscopic evaluation of vitiligo lesions was also performed, and findings were analyzed in relation to serum GNLY levels to determine whether specific dermoscopic features correlated with biomarker expression and disease activity.

### 2.5. Biochemical Analysis

#### 2.5.1. Principles of the Assay

GNLY levels were estimated in serum by an enzyme-linked immunosorbent assay (ELISA) using a commercial kit obtained from Shanghai Sunred Biological Technology Co., Ltd. (Shanghai, China). The GNLY concentration was measured using a double-antibody sandwich ELISA. Samples were added to wells pre-coated with human GNLY monoclonal antibodies, followed by biotin-labeled GNLY antibodies and Streptavidin-HRP to form an immune complex. After incubation and washing to remove unbound components, chromogen solutions A and B were added, producing a blue color that turned yellow with acid. The color intensity was directly proportional to GNLY concentration. A standard curve was generated, and sample concentrations were determined by interpolation from this curve.

#### 2.5.2. Procedures

Before analysis, all plasma and homogenized samples were brought to room temperature and vortexed. The required number of ELISA wells was selected, with unused wells resealed with desiccant and stored at 2–8 °C. Wells were placed in a holding frame, and 50 µL of standard and 40 µL of each control or sample were added to designated wells. Subsequently, 10 µL of GNLY antibody and 50 µL of Streptavidin-HRP were added to all wells. The plate was incubated for 60 min at 37 °C. Following incubation, wells were washed three times by adding 0.4 mL of wash buffer and aspirating the contents. Next, 50 µL each of Chromogen Solution A and B were added, mixed gently, and incubated for 10 min at 37 °C in the dark. Finally, 50 µL of stop solution was added to each well, and absorbance was measured at 450 nm with a reference at 630 nm within 15 min. GNLY concentrations were determined by interpolating from a standard calibration curve generated by plotting absorbance values against known GNLY concentrations.

### 2.6. Statistical Power/Effect Size

We did not calculate sample size before the start of the study, but we calculated effect size as follows. Patients were consecutively recruited from the Dermatology Outpatient Clinic of Tanta University Hospital from January 2022 to August 2023. This consecutive sampling approach was chosen to minimize selection bias. Post hoc effect size and power calculations were performed for the principal comparisons using observed group means and standard deviations. Cohen’s d was calculated for pairwise comparisons, and Cohen’s f for the one-way ANOVA across all four groups. A two-sided α = 0.05 was used. These post hoc calculations were intended to assess the magnitude of observed effects and the achieved power in the present sample.

### 2.7. Statistical Analysis

Statistical analysis was performed using SPSS version 27 (IBM©, Chicago, IL, USA). The Shapiro–Wilk test assessed data normality. Parametric quantitative variables were expressed as mean ± standard deviation (SD) and analyzed using one-way ANOVA followed by Tukey’s post hoc test. Non-parametric quantitative variables were reported as median and interquartile range (IQR), with group comparisons conducted using the Kruskal–Wallis test and pairwise comparisons via the Mann–Whitney U test. Categorical variables were presented as frequencies and percentages, and analyzed using the Chi-square test. Correlations between variables were evaluated using Pearson’s correlation coefficient. A two-tailed *p*-value < 0.05 was considered statistically significant.

## 3. Results

### 3.1. Analysis of Baseline Demographic Data

The demographic and clinical characteristics of the studied groups are presented in Table 1. The median age of participants did not differ significantly between groups (*p* = 0.109), with median values of 14 years (IQR 11–23) in active vitiligo, 17 years (IQR 14–35) in stable vitiligo, 20 years (IQR 10.5–32) in alopecia areata (AA), and 25 years (IQR 22.5–28) in controls. The sex distribution was also comparable (*p* = 0.807), with males representing 40.0%, 48.0%, 53.3%, and 53.3% of the respective groups.

A positive family history was observed in 20% of both active and stable vitiligo patients, whereas none of the AA cases reported a family history (MC = 0.196). The duration of disease differed significantly across groups (*p* < 0.001), being longest in stable vitiligo (median 4 years, IQR 2–7), followed by active vitiligo (median 3 years, IQR 1–7), and shortest in AA (median 0.25 years, IQR 0.08–0.5). Pairwise comparisons showed significant differences between controls and each disease group (p0 < 0.001) as well as between active vitiligo and AA (p2 < 0.001) and stable vitiligo and AA (p3 < 0.001), whereas no significant difference was observed between active and stable vitiligo (p1 = 0.427).

Regarding associated diseases, most patients and all controls had no comorbidities (88.0% in active vitiligo, 96.0% in stable vitiligo, and 100% in both AA and controls; *p* = 0.966). A few cases of diabetes mellitus, Helicobacter pylori infection, and hypertension were reported exclusively among vitiligo patients.

### 3.2. Comparison Between Active Vitiligo and Stable Vitiligo According to the Site and Type of Vitiligo, VIDA, VASI Scores, Dermoscopic Data and Serum GNLY Level

When comparing patients with active and stable vitiligo, several significant differences were observed (Table 2). Regarding the site of lesions, trunk involvement was significantly more frequent in the active vitiligo group compared to the stable group (32.0% vs. 0.0%, *p* = 0.004). In terms of vitiligo type, the acral form was more common among stable vitiligo patients (68.0% vs. 32.0%, *p* = 0.011), whereas the vulgaris type was significantly associated with active vitiligo (32.0% vs. 4.0%, *p* = 0.023).

The VIDA score showed a highly significant difference between groups, with all stable vitiligo patients classified as VIDA 0, while the active group presented with VIDA scores of +2, +3, and +4 (*p* < 0.001). By contrast, the VASI scores did not differ significantly between active and stable vitiligo (median 10 vs. 10, *p* = 0.731).

Dermoscopic examination revealed striking differences. Ill-defined borders (100.0%), satellite lesions (64.0%), and starburst appearance (52.0%) were exclusively observed in active vitiligo, while perifollicular pigmentation (100.0%) and reticulate pigment network (88.0%) predominated in stable disease (*p* < 0.001 for all comparisons).

Finally, serum granulysin (GNLY) levels were significantly lower in the active vitiligo group compared with the stable group [median (IQR): 17.19 (14.16–26.76) vs. 26.34 (23.17–30.90), *p* = 0.005].

### 3.3. Comparison of Serum GNLY Level Between All Studied Groups

Serum GNLY levels showed significant variation among the studied groups (*p* < 0.001). Patients with active vitiligo (26.01 ± 8.37 ng/mL) and those with active alopecia areata (27.05 ± 5.87 ng/mL) had the highest levels, while stable vitiligo patients demonstrated intermediate values (14.10 ± 3.84 ng/mL), and healthy controls showed the lowest levels (6.90 ± 2.08 ng/mL). Pairwise comparisons confirmed that active vitiligo cases had significantly higher GNLY levels compared to stable vitiligo (*p* < 0.001), but were comparable to active alopecia areata (*p* = 0.947). In contrast, stable vitiligo patients had significantly lower GNLY levels than those with active alopecia areata (*p* < 0.001). When analyzed collectively, vitiligo patients (20.06 ± 8.82 ng/mL) exhibited significantly higher levels compared to controls (*p* < 0.001) and differed significantly from alopecia areata cases (*p* = 0.006). Finally, both vitiligo and alopecia areata groups showed markedly elevated GNLY levels compared to controls (*p* < 0.001) as shown in Table 3.

### 3.4. Correlation Between Serum GNLY Level with the Duration of the Disease, Age, VIDA Score and VASI Score in All Vitiligo Cases

There was no significant correlation between serum GNLY level and the age and VASI score of vitiligo cases. There was a significant correlation between serum GNLY level and VIDA score as shown in Table 4.

### 3.5. Relation Between Serum GNLY and Sex and Dermoscopic Data in Vitiligo Cases

There was no significant relation between serum GNLY level and the sex and starburst appearance of vitiligo cases. There was a significant relation between serum GNLY level and ill-defined border, satellite lesions, perifollicular pigmentation, and absent pigment network as shown in Table 5.

### 3.6. Figure Analysis of the Studied Groups

**Figure 1:**
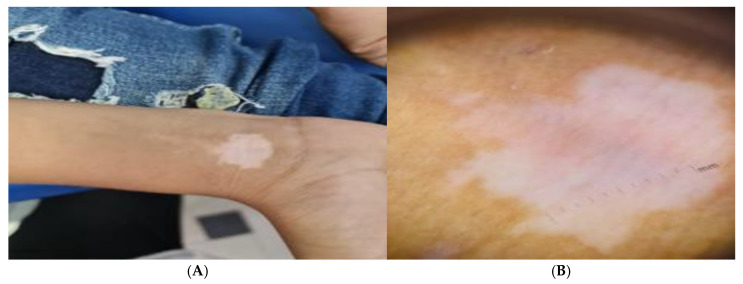
(**A**) Clinical picture of the active acral vitiligo patient showing depigmented patch on the forearm and (**B**) Dermoscopic picture showing absent pigment network.

**Figure 2:**
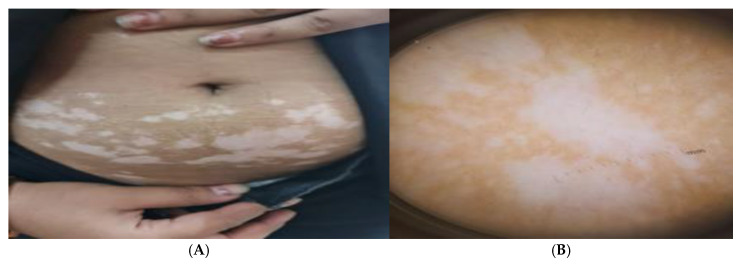
(**A**) Clinical picture of active non-segmental vitiligo patient showing multiple depigmented patches on the trunk and (**B**) Dermoscopic picture of the patient showing a starburst appearance and ill-defined border.

Patients with active vitiligo showed depigmented patch on the forearm (Figure 1A), and Dermoscopic picture showed absent pigment network (Figure 1B), meanwhile patients with active non-segmental vitiligo showed multiple depigmented patches on the trunk (Figure 2A), and Dermoscopic picture of the patient showed a starburst appearance and ill-defined border (Figure 2B).

**Figure 3:**
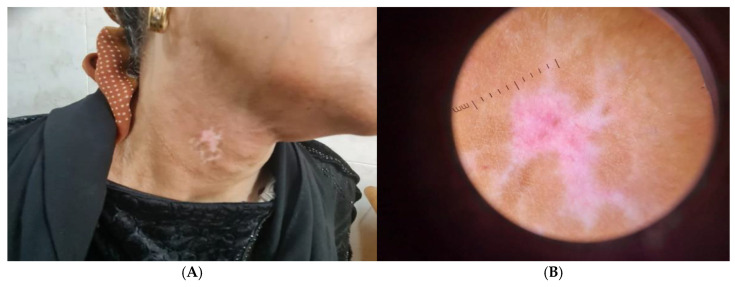
(**A**) Clinical picture of stable segmental vitiligo patient showing depigmented patch on neck and (**B**) Dermoscopic picture showing erythema and telangiectasia.

In addition, patients with stable segmental vitiligo showed depigmented patch on neck (Figure 3A), and Dermoscopic picture revealed erythema and telangiectasia (Figure 3B).

**Figure 4:**
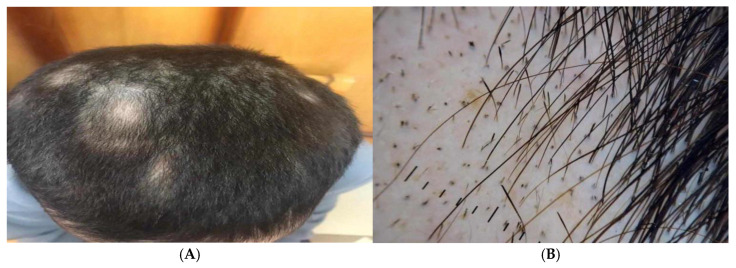
Male patients showed (**A**) Clinical picture showing multiple patches of hair loss and (**B**) Dermoscopic picture showing yellow dots, black dots, short regrowing hair, and exclamation mark.

**Figure 5:**
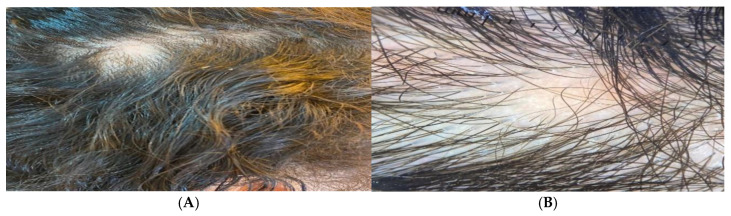
Female patient showing (**A**) Clinical picture showing a patch of hair loss and (**B**) Dermoscopic picture showing yellow dots showing intralesional erythema, telangiectasia, and leukotrichia.

Alopecia areata patients showed multiple patches of hair loss (Figure 4A and Figure 5A), and Dermoscopic picture showed yellow dots, black dots, short regrowing hair, and exclamation mark (Figure 4B and Figure 5B).

### 3.7. Post Hoc Effect Size Analysis

Post hoc effect size estimates demonstrated very large effects for GNLY differences: active vitiligo versus stable vitiligo, Cohen’s d = 1.83 (pooled SD ≈ 6.51); active alopecia areata versus controls, Cohen’s d = 4.58 (pooled SD ≈ 4.40); combined vitiligo (n = 50) versus controls, Cohen’s d = 2.26 (pooled SD ≈ 5.83). The overall one-way ANOVA effect size was Cohen’s f = 1.39. Using the observed effects and the sample size (total N = 80), the post hoc power for the overall ANOVA and for the principal pairwise comparisons exceeded 99% (α = 0.05). These post hoc results indicate the present sample was well-powered to detect the observed differences.

## 4. Discussion

The present study investigated serum granulysin (GNLY) levels in patients with vitiligo and alopecia areata (AA), two autoimmune dermatological conditions that share overlapping immunological features. Our results demonstrate that GNLY levels are significantly elevated in patients with active vitiligo and AA compared with stable vitiligo and healthy controls. Importantly, GNLY levels correlated with established clinical indices, including the Vitiligo Disease Activity (VIDA) score and dermoscopic indicators of activity, supporting its potential as a biomarker for disease monitoring. These findings extend previous observations of immune dysregulation in vitiligo and AA and underscore the potential role of GNLY in the cytotoxic processes that underlie these conditions [12,25].

In vitiligo, our findings revealed that patients with active disease exhibited substantially higher GNLY levels than those with stable disease. Ono et al. [26] found that circulating GNLY levels were significantly higher in AA patients than in healthy individuals. This distinction is clinically meaningful, as one of the greatest challenges in vitiligo management is differentiating between progressive and stable disease stages. Elevated GNLY was positively associated with VIDA scores, a widely accepted measure of vitiligo activity [27], and with dermoscopic features such as ill-defined lesion borders, satellite lesions, and starburst appearances—all of which signify ongoing melanocyte destruction [28,29]. Conversely, lower GNLY levels were observed in association with features typically linked to stability, such as perifollicular pigmentation and reticulate pigment network [30]. This aligns with findings from Purnima et al. [31], Jha et al. [24] and Chuh AA and Zawar V. [32], who identified dermoscopic features such as micro-Koebner phenomenon, satellite lesions, micro-confetti, and starburst patterns as strong indicators of vitiligo activity. Other features—like perifollicular pigmentation or depigmentation, absent or faint pigment network, marginal pigmentation, and trichrome appearance—were less conclusive for determining disease activity or stability, and often appeared concurrently within the same lesion. Chandrashekar [33] also noted dermoscopic signs associated with both active and stable vitiligo. Features linked to stability and repigmentation included marginal and perifollicular hyperpigmentation, reticular pigmentation, and marginal reticular pigmentation. Our findings should be considered within the expanding literature on biomarkers in autoimmune dermatology. Several studies have demonstrated that plasma and lesional levels of IFN-γ, IFN-inducible chemokines (CXCL9/CXCL10) and cytotoxic effectors such as granzyme B correlate with vitiligo activity, supporting a systemic readout of local immune events [34,35]. Taken together, these findings suggest that GNLY not only reflects disease presence but also mirrors the degree of immunological activity, thereby providing an objective complement to clinical and dermoscopic evaluation.

In the case of AA, our study similarly found elevated GNLY levels in patients compared with healthy controls. In cases of acute AA, GNLY levels positively correlated with disease severity. Additionally, GNLY-expressing cytotoxic cells were identified surrounding hair follicles in both acute and chronic AA cases [36]. In contrast, Maraee et al. [37] observed a significant positive correlation between serum GNLY levels and the recurrence, duration, and severity of alopecia. Their findings suggest that GNLY may serve as a novel and valuable biomarker for assessing disease activity and prognosis during the acute phase of AA. Although the AA subgroup was relatively small, the results are biologically plausible given the known pathogenesis of AA. CD8^+^ cytotoxic T lymphocytes and natural killer (NK) cells are central to hair follicle destruction in AA [38,39], and both cell populations are well-established sources of GNLY [26]. The elevation of GNLY in our cohort is therefore consistent with heightened cytotoxic activity at the hair follicle level [26]. This observation supports the hypothesis that GNLY may serve as a general indicator of autoimmune cytotoxic responses in cutaneous tissues, rather than being disease-specific. Future larger studies in AA cohorts are warranted to confirm these findings and to explore the potential of GNLY as a biomarker in clinical trials assessing novel therapies for AA. In AA, systematic reviews have reported associations between disease and elevated inflammatory mediators (for example IL-6 and CRP) as well as alterations in other immune parameters [40,41]. These data suggest overlapping and disease-specific biomarker patterns. Prior reports specifically implicate GNLY as elevated in active AA [36] and, supporting our results and suggesting that GNLY may sit alongside IFN-driven chemokines and cytotoxic molecules as an indicator of immune-mediated tissue damage. However, most evidence to date is correlative and heterogeneous in sample size and methodology.

The shared elevation of GNLY in both vitiligo and AA further reinforces their immunological overlap. Both conditions are considered autoimmune diseases driven by effector T cells and NK cells, with tissue-specific destruction targeting melanocytes in vitiligo and hair follicle keratinocytes in AA [9,42]. GNLY is a key cytotoxic protein released from the granules of activated CD8^+^ T cells and natural killer (NK) cells, both of which are central effector cells in autoimmune skin diseases [43]. In vitiligo, CD8^+^ T cells infiltrate the epidermis and target melanocytes [44], while in AA, these cells accumulate around the hair bulb and attack melanocyte-associated follicular cells [45]. Upon activation, both CD8^+^ T cells and NK cells secrete perforin and granzymes along with GNLY [46]. Perforin facilitates pore formation in the target cell membrane, allowing entry of granzymes, whereas GNLY exerts direct cytolytic effects by disrupting cell membranes and inducing mitochondrial apoptosis [47]. Additionally, GNLY has antimicrobial and proinflammatory properties, further amplifying local tissue injury and perpetuating immune activation [48]. Thus, elevated circulating GNLY levels reflect ongoing CD8^+^ T-cell and NK-cell–mediated cytotoxicity, supporting its role as both a mechanistic effector and a biomarker of disease activity in vitiligo and alopecia areata [49]. By demonstrating elevated systemic GNLY in both diseases, our study adds weight to the growing body of evidence that these two conditions may represent different clinical manifestations of a shared autoimmune disease. This insight could have broader implications for understanding other autoimmune skin disorders and supports the exploration of GNLY as a biomarker across a spectrum of dermatological conditions [50].

From a clinical perspective, our findings highlight several potential applications of GNLY measurement. In vitiligo, identifying patients with active disease is crucial for timely initiation of immunosuppressive or immunomodulatory treatment, as interventions are most effective before complete melanocyte loss occurs [51]. GNLY levels may provide an additional tool for stratifying patients, guiding treatment decisions, and monitoring therapeutic response [12]. In AA, GNLY could potentially serve a similar role in identifying active inflammation within the hair follicle unit, aiding in treatment selection and follow-up [36]. Beyond disease monitoring, the observation that GNLY is consistently elevated in both vitiligo and AA raises the possibility that GNLY itself may represent a therapeutic target. Blocking GNLY or modulating its activity could theoretically attenuate cytotoxic immune responses, although this remains speculative and requires experimental validation [52,53]. The measurement of serum GNLY holds promise as a clinically relevant biomarker in autoimmune skin diseases. Its role as a cytotoxic effector molecule links it directly to disease pathophysiology, and its detectability in peripheral blood provides a minimally invasive tool for patient assessment. In clinical practice, GNLY could complement existing clinical scores by helping to identify active disease, monitor treatment response, and potentially stratify patients who are more likely to benefit from targeted immunomodulatory therapies. Although additional validation in larger and longitudinal cohorts is required, GNLY may serve as a step toward more personalized management of vitiligo and alopecia areata.

Despite the strengths of our study, several limitations must be acknowledged. First, the sample size was modest, particularly for the AA subgroup, which reduces the statistical power of subgroup analyses and limits the generalizability of the findings. Second, we did not perform longitudinal follow-up to assess fluctuations in serum GNLY levels in relation to changes in disease activity over time. As a result, we could not determine whether GNLY levels vary dynamically with disease progression or remission. Future prospective studies with serial sampling are warranted to clarify the temporal relationship between GNLY expression and clinical activity in vitiligo and alopecia areata. Future prospective studies are needed to confirm whether GNLY levels decrease in response to effective therapy or rise prior to clinical relapse. Third, it is important to distinguish between correlation and causation in interpreting our findings. Elevated serum GNLY reflects the heightened activity of cytotoxic CD8^+^ T cells and NK cells, and thus correlates with disease activity in vitiligo and alopecia areata. However, the present study does not establish GNLY as a direct pathogenic driver, but rather as a surrogate marker of immune-mediated cytotoxicity. From a translational perspective, GNLY may still hold therapeutic relevance. While direct inhibition of GNLY has not yet been explored in clinical practice, modulation of the pathways leading to its release (e.g., targeting CD8^+^ T-cell or NK-cell activation, perforin/granzyme signaling, or IFN-γ/IL-15 pathways) could indirectly reduce GNLY-mediated cytotoxicity. Future mechanistic studies are warranted to determine whether GNLY is merely a biomarker or could represent a potential therapeutic target in autoimmune skin diseases. Fourth, our study did not include lesional or perilesional tissue samples, which would have provided valuable information on local GNLY expression relative to systemic levels. Finally, some of the clinical and dermoscopic images presented in the manuscript may appear familiar to dermatology specialists, as they were intended primarily for illustration of activity patterns rather than to introduce novel diagnostic features.

Future research should aim to address these limitations. Multi-center studies with larger and more diverse cohorts are required to validate GNLY as a reliable biomarker across populations. Longitudinal studies will be particularly important to clarify the dynamic relationship between GNLY and disease activity over time, as well as its responsiveness to treatment. Mechanistic investigations at the cellular and molecular levels should explore whether GNLY contributes directly to melanocyte or follicular cell destruction, or whether it simply reflects immune activation. Finally, comparative studies assessing GNLY alongside other emerging biomarkers in autoimmune dermatology could clarify its specificity and additive value within biomarker panels.

## 5. Conclusions

In summary, this study demonstrated that serum GNLY levels are significantly elevated in patients with vitiligo and alopecia areata compared with healthy controls, with higher levels observed in active disease states. In vitiligo, GNLY showed a strong positive correlation with VIDA scores, underscoring its potential as a biomarker of disease activity. Similarly, patients with alopecia areata exhibited markedly increased GNLY levels, supporting the role of cytotoxic immune responses in the pathogenesis of both disorders. Importantly, the shared elevation of GNLY across these autoimmune conditions highlights a possible common immunopathogenic pathway, suggesting that GNLY may serve not only as a disease marker but also as a potential therapeutic target.

Clinically, these findings may help guide disease monitoring and support the early identification of active disease phases, which are crucial for timely intervention. Nevertheless, our study is limited by its relatively small sample size, the cross-sectional design, and the absence of longitudinal follow-up to determine GNLY dynamics over time. Future research should aim to validate these results in larger, multicentre cohorts, evaluate GNLY changes in response to treatment, and explore its utility as a predictive marker for disease progression or relapse.

Taken together, our findings provide new insights into the role of GNLY in autoimmune dermatological diseases and support its further investigation as a biomarker and potential therapeutic target in vitiligo and alopecia areata.

## Figures and Tables

**Table 1:** Comparison between the four studied groups according to demographic data and associated disease.

Variable	Active Vitiligo (n = 25)	Stable Vitiligo (n = 25)	Active AA (n = 15)	Controls (n = 15)	*p*-Value
Age, years (median [IQR])	14 (11–23)	17 (14–35)	20 (10.5–32)	25 (22.5–28)	0.109
Sex, n (%)					0.807
• Male	10 (40.0)	12 (48.0)	8 (53.3)	8 (53.3)	
• Female	15 (60.0)	13 (52.0)	7 (46.7)	7 (46.7)	
Family history, n (%)	5 (20.0)	5 (20.0)	0 (0.0)	–	MC = 0.196
Duration of disease, years (median [IQR])	3 (1–7)	4 (2–7)	0.25 (0.08–0.5)	–	<0.001 *
	p0 < 0.001 *, p1 = 0.427, p2 < 0.001 *, p3 < 0.001 *				
Associated diseases, n (%)					0.966
• None	22 (88.0)	24 (96.0)	15 (100.0)	15 (100.0)	
• Diabetes mellitus	1 (4.0)	1 (4.0)	0 (0.0)	0 (0.0)	
• Helicobacter pylori	1 (4.0)	0 (0.0)	0 (0.0)	0 (0.0)	
• Hypertension	1 (4.0)	0 (0.0)	0 (0.0)	0 (0.0)	

Data are presented as median and interquartile range (IQR) or frequency (%). * Significant at *p* < 0.05. MC = Monte Carlo test. p0: comparison between controls and each other group. p1: comparison between active vitiligo and stable vitiligo. p2: comparison between active vitiligo and active alopecia areata. p3: comparison between stable vitiligo and active alopecia areata. AA = alopecia areata.

**Table 2:** Comparison between active and stable vitiligo groups.

Variable	Active Vitiligo (n = 25)	Stable Vitiligo (n = 25)	*p*-Value
Site of lesions, n (%)			
• Head and neck	12 (48.0)	7 (28.0)	0.145 ^1^
• Trunk	8 (32.0)	0 (0.0)	0.004 *^2^
• Extremities	10 (40.0)	8 (32.0)	0.556 ^1^
• Acral	13 (52.0)	17 (68.0)	0.248 ^1^
Type of vitiligo, n (%)			
• Segmental	2 (8.0)	4 (16.0)	0.667 ^2^
• Non-segmental	23 (92.0)	21 (84.0)	0.384
– Acral	8 (32.0)	17 (68.0)	0.011 *^1^
– Acrofacial	7 (28.0)	3 (12.0)	0.157 ^1^
– Vulgaris	8 (32.0)	1 (4.0)	0.023 *^2^
VIDA score, n (%)			<0.001 *^3^
• 0	0 (0.0)	25 (100.0)	
• 2+	6 (24.0)	0 (0.0)	
• 3+	13 (52.0)	0 (0.0)	
• 4+	6 (24.0)	0 (0.0)	
VASI (%) (median [IQR])	10 (10–10)	10 (5–15)	0.731 ^4^
Dermoscopic findings, n (%)			<0.001 *^2^
• Ill-defined border	25 (100.0)	0 (0.0)	<0.001 *^2^
• Satellite lesions	16 (64.0)	0 (0.0)	<0.001 *^2^
• Starburst appearance	13 (52.0)	0 (0.0)	<0.001 *^2^
• Perifollicular pigmentation	2 (8.0)	25 (100.0)	<0.001 *^2^
• Reticulate pigment network	5 (20.0)	22 (88.0)	0.001 *^2^
Serum GNLY (ng/mL) (median [IQR])	17.19 (14.16–26.76)	26.34 (23.17–30.90)	0.005 *^4^

1. Chi-square test. 2. Fisher’s exact test. 3. Monte Carlo correction. 4. Mann–Whitney U test. Data are presented as median (IQR) or frequency (%). *p* < 0.05 considered statistically significant. VIDA = Vitiligo Disease Activity score; VASI = Vitiligo Area Scoring Index; GNLY = granulysin; * = it refers to the significance.

**Table 3:** Comparison of serum GNLY level (between vitiligo, AA, and control), (vitiligo and control) and between (vitiligo AA, and control).

Group	Serum GNLY (ng/mL), Mean ± SD	Overall *p*-Value (ANOVA)
Active vitiligo (n = 25)	26.01 ± 8.37	<0.001 *
Stable vitiligo (n = 25)	14.10 ± 3.84	
Active AA (n = 15)	27.05 ± 5.87	
Controls (n = 15)	6.90 ± 2.08	
Pairwise comparison		***p*-value**
Active vitiligo vs. Stable vitiligo		<0.001 *
Active vitiligo vs. Active AA		0.947
Stable vitiligo vs. Active AA		<0.001 *
Vitiligo (all, n = 50) vs. Controls (n = 15)		<0.001 *
Vitiligo (all, n = 50) vs. Active AA		0.006 *
Active AA vs. Controls		<0.001 *

Data are presented as mean ± SD. ANOVA with post hoc pairwise comparisons was used. *: Statistically significant at *p* ≤ 0.05. GNLY = granulysin; AA = Alopecia areata.

**Table 4:** Correlation between serum GNLY level with the duration of the disease, age, VIDA score and VASI score in all vitiligo cases.

Factor	Serum GNLY Level (ng/mL)	
	r	*p*
Duration (years)	−0.267	0.061
Age (years)	−0.160	0.268
VIDA	0.837	<0.001 *
VASI (%)	0.258	0.070

r: Pearson coefficient, *: Statistically significant at *p* ≤ 0.05; VIDA: Vitiligo Disease Activity Score; VASI: Vitiligo Area Scoring Index; GNLY: granulysin level.

**Table 5:** Relation between Serum GNLY and sex and Dermoscopic data in all vitiligo cases.

Factor		N	GNLY (ng/mL)	*p*
Sex	Male	22	19.20 ± 8.48	0.550
	Female	28	20.73 ± 9.17	
Dermoscopic Data				
Ill-defined border		28	24.65 ± 8.95	<0.001 *
Satellite lesion		21	23.73 ± 8.81	0.011 *
Starburst appearance		17	21.69 ± 8.79	0.354
Per follicular pigmentation		16	15.46 ± 6.41	0.004 *
Absent pigment network		27	16.20 ± 6.86	0.001 *

Data are presented as mean ± SD. *: Statistically significant at *p* ≤ 0.05; GNLY: granulysin level.

## Data Availability

Upon an appropriate request, any data can be obtained from the corresponding author.
